# The Dynamics of Treg/Th17 and the Imbalance of Treg/Th17 in *Clonorchis sinensis*-Infected Mice

**DOI:** 10.1371/journal.pone.0143217

**Published:** 2015-11-23

**Authors:** Chao Yan, Bei-Bei Zhang, Hui Hua, Bo Li, Bo Zhang, Qian Yu, Xiang-Yang Li, Ying Liu, Wei Pan, Xiang-Ye Liu, Ren-Xian Tang, Kui-Yang Zheng

**Affiliations:** Department of Pathogenic Biology and Immunology, Laboratory of Infection and Immunity, Xuzhou Medical College, Xuzhou 221004, Jiangsu Province, China; Wayne State University, UNITED STATES

## Abstract

Clonorchiasis, caused by the liver fluke *Clonorchis sinensis*, is a chronic parasitic infection regulated by T cell subsets. An imbalance of CD4^+^CD25^+^ Foxp3^+^regulatory T (Treg) and interleukin (IL)-17-secreting T cells (Th17) may control inflammation and play an important role in the pathogenesis of immune evasion. In the present study, we assessed the dynamics of Treg/Th17 and determined whether the Treg/Th17 ratio is altered in *C*. *sinensis*-infected mice. The results showed that the percentages of splenic Treg cells in CD4^+^ T cells were suppressed on day 14 post-infection (PI) but increased on day 56 PI, while Th17 cells were increased on day 56 PI compared with normal control (NC) mice. The Treg/Th17 ratio steadily increased from day 28 to day 56 PI. The hepatic levels of their specific transcription factors (Foxp3 for Treg and RORγt for Th17) were increased in *C*. *sinensis*-infected mice from day 14 to 56 PI, and significantly higher than those in NC mice. Meanwhile, serum levels of IL-2 and IL-17 were profoundly increased in *C*. *sinensis*-infected mice throughout the experiment; while the concentrations of IL-6 and transforming growth factor β_1_ (TGF-β_1_) peaked on day 14 PI, but then decreased on day 28 and 56 PI. Our results provide the first evidence of an increased Treg/Th17 ratio in *C*. *sinensis*-infected mice, suggesting that a Treg/Th17 imbalance may play a role in disease outcomes of clonorchiasis.

## Introduction

Clonorchiasis is a disease affecting about 15 million people; it is caused by *Clonorchis sinensis* that is widely distributed in east and southeast Asia including Korea, China, Russia, and Vietnam [1–2]. People can become infected by ingesting raw or undercooked freshwater fish containing infective *C*. *sinensis* metacercariae. Digestive juice in the duodenum stimulate the swallowed metacercariae to excyst, and the fresh *C*. *sinensis* juveniles move to the intrahepatic bile duct where they grow into adult worms and can live for decades [3–4]. Chronic *C*. *sinensis* infection can result in pathological changes such as biliary duct proliferation, inflammation around the porta vein, periductal fibrosis, and cirrhosis [5–6]. *C*. *sinensis* accompanied by *Opisthorchis viverrini* is considered a biological carcinogen that can potently lead to cholangiocarcinoma [7–8]; however, the mechanism underlying the development of these pathological changes is not clear.

CD4^+^CD25^+^Foxp3^+^regulatory T (Treg) cells play a vital role in maintaining self-tolerance, immune balance, preventing allergies, and confining inflammatory responses to infectious diseases [9–10]. Autoimmunity and inflammatory diseases may occur due to defective Treg cell functioning; this cell type can potently suppress effector T cells and other immune cells through cell-cell contact-dependent immunosuppression or the release of anti-inflammatory factors such as transforming growth factor (TGF-β_1_) and interleukin (IL)-10 [9–12]. Treg cells are a key regulator of immune responsiveness that promote parasite survival (e.g., *Trichuris muris*) and control worm-induced pathology [13–15].

A new subset of CD4^+^T helper cells that secrete IL-17 has recently been identified and termed Th17 cells. These cells are distinct from Th1 or Th2 cells and responsible for the immunopathology of autoimmune diseases including experimental autoimmune encephalomyelitis (EAE), which is a widely used mouse model of multiple sclerosis [16–17]. Th17 cell may also play an important role in the immunopathology of infectious diseases and in host defense against certain extracellular pathogens [18–19]. Th17 cells are closely related to Treg cells as they originate from the same naïve CD4^+^ T cell precursors, and both require TGF-β_1_ for differentiation. For example, following induced by a high dose of TGF-β_1_ combined with IL-2, naïve CD4^+^ T cells can differentiate into Treg cells with the requirement of forkhead family protein 3 (Foxp3); naïve CD4^+^T cells can also develop into Th17 cells and express RAR-related orphan receptor gamma (RORγt) under the influence of a low dose of TGF-β_1_ together with IL-6 [20–23].

Increasing evidences demonstrate that an imbalance between Th17 and Treg is associated with the development of various infectious diseases including parasitical diseases, indicating a potential role for a Treg/Th17 imbalance in parasitic pathogenesis [18,24–26]. For example, the ratio of Treg to Th17 is decreased in pregnant mice infected with *Toxoplasma gondii*, which may contribute to parasite-induced embryo loss [24]. The increased Th17/Treg ratio may also be involved in the pathogenesis of human schistosomiasis [19]. Little is known about whether and when the Treg/Th17 balance is disturbed in *C*. *sinensis* infection or if it contributes to clonorchiasis pathogenesis. Here, we measured the dynamic changes in Treg/Th17 and Treg/Th17-related cytokine levels in *C*. *sinensis*-infected mice.

## Material and Methods

### Parasites

*C*. *sinensis* metacercariae were prepared as previously described [27]. Briefly, fresh fish naturally infected with *C*. *sinensis* metacercariae were homogenized and digested through artificial gastric juice (0.7% pepsin in 1% HCl solution, pH 2.0) for 12 h, and the metacercariae were isolated under a dissecting microscope. The prepared metacercariae were stored in Alsever’s solution at 4°C until required.

### Animals, infection, and sampling

We purchased 6–8-week-old female BALB/c mice from the Shanghai Experimental Animal Center, Chinese Academy of Sciences (Shanghai, China); they were housed under specific pathogen-free conditions with a 12-h light/dark cycle and provided with food and water *ad libitum*. The study protocols complied with the Guide for the Care and Use of Laboratory Animals of the Ministry of Health, China and were approved by the Animal Ethics Committee of Xuzhou Medical College (No. SCXK<SU>2014–0003). All efforts made were to minimize animal suffering.

Mice received 45 *C*. *sinensis* metacercariae orally. *C*. *sinensis*-infected animals (n = 24, 8 mice for each time-point) and normal control (NC) animals (n = 8) were euthanized on days 14, 28, and 56 post-infection (PI). Partial left lobes of the livers from all mice were fixed in 10% neutral buffered formalin and embedded in paraffin for histological examination. Serum from each mouse was also collected and stored at -80°C to evaluate Treg/Th17-related cellular cytokines.

### Examination of *C*. *sinensis* eggs

Feces from all infected mice were screened to detect *C*. *sinensis* eggs microscopically from day 25 to 56 PI. Briefly, feces collected from each mouse at these time-points were dissolved for 12 h in 10% NaOH solution, subsequently smeared onto labeled slides to examine *C*. *sinensis* eggs under a microscope (Olympus, Tokyo, Japan). All samples were examined at least three times.

### Flow cytometry

Flow cytometry was performed as described elsewhere [28–29]. In brief, for Th17 detection, approximately 10^6^ splenocytes were prepared and incubated with PMA (20 ng/m) and ionomycin (1 μg/mL) in the presence of monensin (2 mmol/ml, Sigma-Aldrich, St. Louis, MO, USA) for 4 h (37°C, 5% CO_2_). The cells were subjected to allophycocyanin (APC)-anti-CD3 and phycoreythrin (PE)-Cy5-anti-CD4 staining at 4°C for 30 minutes in the dark. After surface staining, the cells were fixed in paraformaldehyde and permeabilized in Perm/Fix solution according to the manufacturer’s instructions (eBioscience, San Diego, CA, USA) and then intracellularly stained with anti-mouse PE-IL-17. To detect Treg cells, the cell suspensions were stained with APC-anti-CD3, PE-cy5-anti-CD4, and fluorescein isothiocyanate (FITC)-anti-CD25 at 4°C for 30 minutes; treated with eBioscience Perm/Fix mixture; and incubated with anti-mouse Foxp3 antibodies according to the manufacturer’s instructions (eBioscience). All of the stained cells were evaluated using a flow cytometer (FACSCanto II; BD Biosciences, Franklin Lakes, NJ, USA), and the data were analyzed using Diva software (BD Biosciences).

### Western-blot assay

Liver sections from mice were crushed in liquid nitrogen and then lysed in NP 40 lysis buffer (50 mM Tris-Cl, pH 8.0, 100 mM NaCl, 5 mM MgCl_2_, 0.5% (v/v) Nonidet P-40, protease inhibitor cocktail, Beyotime Biotech, Beijing, China) on ice for 30 min. The protein concentration was determined using a BCA Protein Assay Kit (Beyotime Biotech, Beijing, China) following the manufacturer’s procedure. Samples were run on a 10% SDS polyacrylamide gel, transferred to a PVDF membrane, and immunoblotted with primary antibodies Foxp3 (1:2000 dilutions, proteintech, Chicago, USA), RORγt (1:2000 dilutions, proteintech, Chicago, USA). After incubation with secondary antibodies (1:2000, Sigma, San Diego, CA, USA), the signal was detected using the Immun-Star Western Chemiluminescence kit (BioRad, Hercules, CA, USA), quantified using the ChemiDoc XRS Imager and analyzed with the software Image Lab from Bio-Rad (BioRrad, Hercules, CA, USA). Tublin was used as endogenous control and the data were normalized by Tublin levels.

### Enzyme-linked immunosorbent assay (ELISA) and cytometric bead array (CBA)

The serum concentration of TGF-β_1_ was measured using commercial available ELISA kits according to the manufacturer’s instructions (eBioscience). CBAs were performed with a FACSCanto II flow cytometer to evaluate the serum concentrations of IL-17A, IL-6, IL-10, and IL-2. Cytometric data were analyzed using FCAP Array software v 3.0 (BD Biosciences). The concentrations of TGF-β_1_, IL-17A, IL-6, IL-10, and IL-2 were calculated using standard curves.

### Histological examination

The left lobes of the liver from each mouse were fixed and embedded in paraffin, and 4-μm serial sections were prepared. Histological changes were examined by hematoxylin and eosin (H&E) staining, and collagen fibers were stained with Masson’s trichrome.

### Determination of hydroxyproline content

Collagen was determined by estimation of the hydroxyproline content, an amino acid characteristic of collagen. The lysates were used to measure hydroxyproline content using commercially available kits according to the manufacturer’s instructions (Jiancheng Institute of Biotechnology, Nanjing, China).

### Statistical analysis

Data were analyzed using SPSS (version 16.0, SPSS Inc., Chicago, IL, USA) and GraphPad software (GraphPad, San Diego, CA, USA). The results are shown as mean ± standard error of the mean (SEM); the data were assessed for equal variance and were log transformed if required. Comparisons between groups were performed using one-way analyses of variance (ANOVAs) with Tukey’s post-hoc tests when equal variances were assumed. Correlations were calculated using Pearson’s correlation. Differences were considered statistically or significantly different when *P*<0.05 or *P*<0.01, respectively.

## Results

### Examination of *C*. *sinensis* eggs

*C*. *sinensis* eggs were microscopically detected in feces obtained from all treated mice (except for 8 mice sampled on day 14 PI). Twelve out of 16 mice (75%) were *C*. *sinensis*-egg positive during examination.

### The Treg/Th17 ratio was increased in *C*. *sinensis*-infected mice

**Fig 1:**
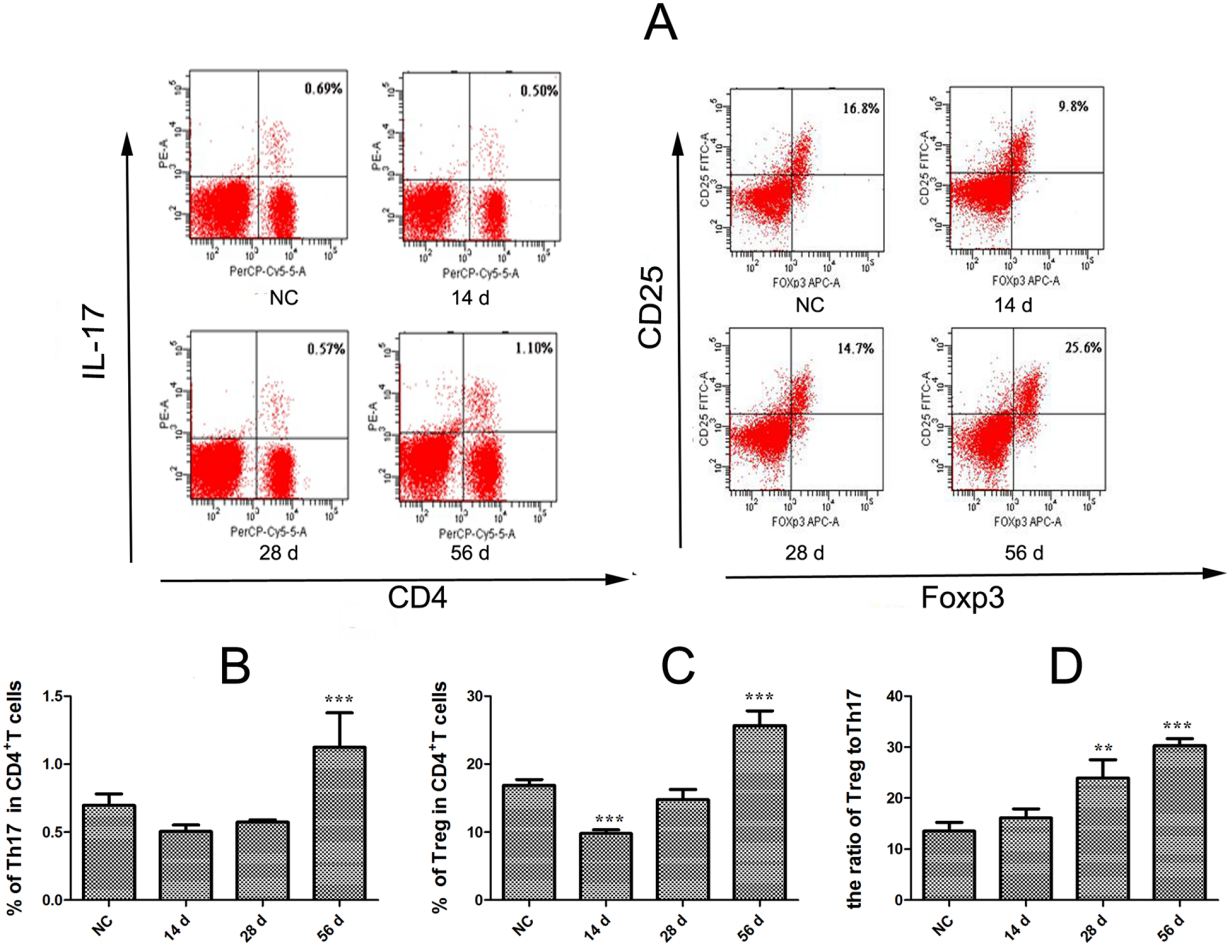
Quantitative changes in splenic Treg and Th17 cell percentages and the relationship between Treg and Th17 subsets. Single-cell spleen suspensions from *C*. *sinensis*-infected and control mice (NC) were used to analyze Th17 and CD4^+^CD25^+^Foxp3^+^regulatory T cell percentages among CD4^+^ T cells by flow cytometry (**A**). Representative blots of IL-17^+^ and CD25^+^Foxp3^+^cells in splenic CD4^+^cell populations. **B** and **C** show quantitative changes in splenic Treg and Th17 cell percentages in CD4^+^T cells. The Treg/Th17 ratio was calculated (**D**). Data are expressed as mean ± S.E.M. Asterisks indicate statistically significant differences between infected and NC mice, **P<0.01, ***P<0.001, compared with normal control mice.

As shown in Fig 1, there was no statistical difference in the Th17 percentage of CD4^+^ T cells on day 14 PI, compared to the NC group (*P*>0.05, Fig 1B), but the percentage of Treg was significantly lower on day 14 PI, compared to the NC group (*P*<0.05, Fig 1C). However, the frequencies of Th17 and Treg in CD4^+^ T cells steadily increased as the infection developed, and significant differences were observed on day 56 PI, compared with the control group (*P*<0.001). There were similar trends in the percentages of Treg and Th17 in total splenic cells (data not shown). The dynamic changes in Treg and Th17 subsets were further explored by calculating the Treg/Th17 ratio. As shown in Fig 1D, the ratio gradually increased as the infection developed, and there were significant differences in 28 and 56 PI. compared with the control group (Fig 1D, *P*<0.01).

### Changes in Foxp3 and RORγt expression in the livers of *C*. *sinensis*-infected mice

**Fig 2:**
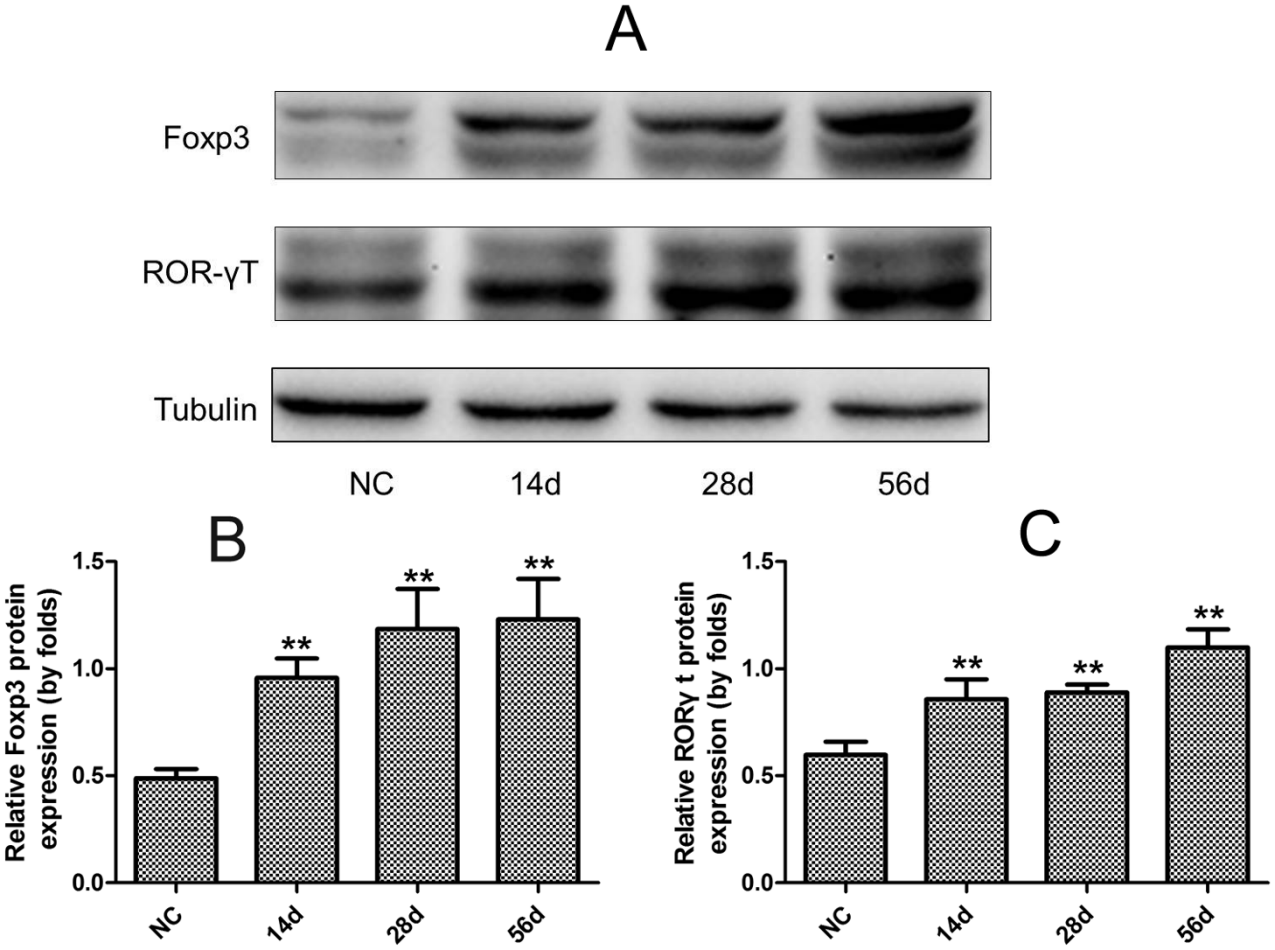
Hepatic expression levels of Foxp3 and RORγt in the mice infected by *Clonorchis sinenis*. Liver sections from *C*. *sinesis*-infected and NC mice were homogenized; and the expression levels of RORγt (**A**) and Foxp3 (**B**) were determined by western-blot. Data are expressed as mean ± S.E.M. Asterisks indicate statistically significant differences between infected and NC mice; **P<0.01, compared with normal control mice.

To further investigate dynamic Treg/Th17 changes in the livers of *C*. *sinensis*-infected mice, we measured expression levels of their specific master regulators (Foxp3 [Treg] and RORγt [Th17]) by western-blot (Fig 2). The results showed similar tendencies for dynamic Foxp3 and RORγt expression levels in the livers of *C*. *sinensis*-infected mice, which indicated that the relative levels of both Foxp3 and RORγt were significant higher than those in the NC group from the day of 14 PI to 56 PI (*P*<0.05, Fig 2B and 2C) and their expression levels in *C*. *sinensis*-infected mice showed the increased tendencies as the infection developed.

### Dynamic changes in Th17 and Treg-related cytokines in *C*. *sinensis*-infected mice

**Fig 3:**
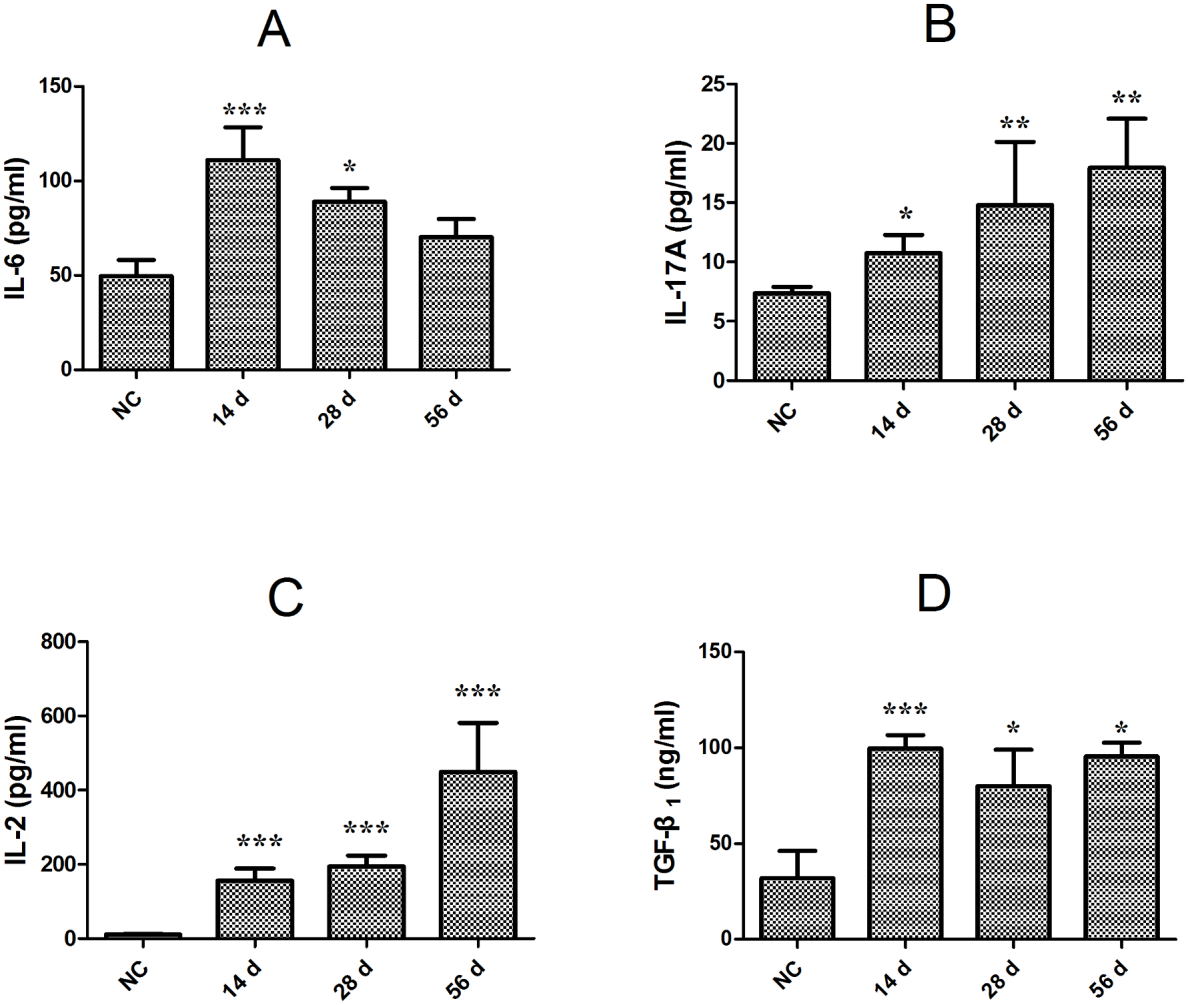
Kinetics of serum IL-17A, IL-6, IL-2, and TGF-β_1_. Serum from each mouse was screened with cytometric bead array (CBA) to detect IL-6 (**A**), IL-17A (**B**), and IL-2 (**C**); enzyme-linked immunosorbent assays (ELISA) was used to measured TGF-β_1_ (**D**). Data are expressed as mean ± S.E.M. Asterisks indicate statistically significant differences between infected mice and NC mice at a particular time point, *P<0.05, **P<0.01, and ***P<0.001, compared with normal control mice.

To further explore the molecular response to the Treg/Th17 imbalance, we measured the Th17- and Treg-related cytokine response to *C*. *sinensis*, including IL-17, IL-6, IL-2, and TGF-β_1_. As demonstrated in Fig 3, IL-17 and IL-2 concentrations markedly increased in infected mice compared with NC mice (Fig 3B and 3C, *P*<0.01). However, the serum levels of IL-6 peaked on day 14 PI. (*P*<0.01 vs. NC) and subsequently dropped to the baseline level as the infection developed (Fig 3A). The serum concentration of TGF-β_1_ in *C*. *sinensis*-infected mice was highest on day 14 PI. (*P*<0.01). Thereafter it decreased, but there were statistical differences between *C*. *sinensis*-infected mice and NC mice (*P*<0.05, Fig 3D).

### Histopathology and collagen depositions of liver tissues

**Fig 4:**
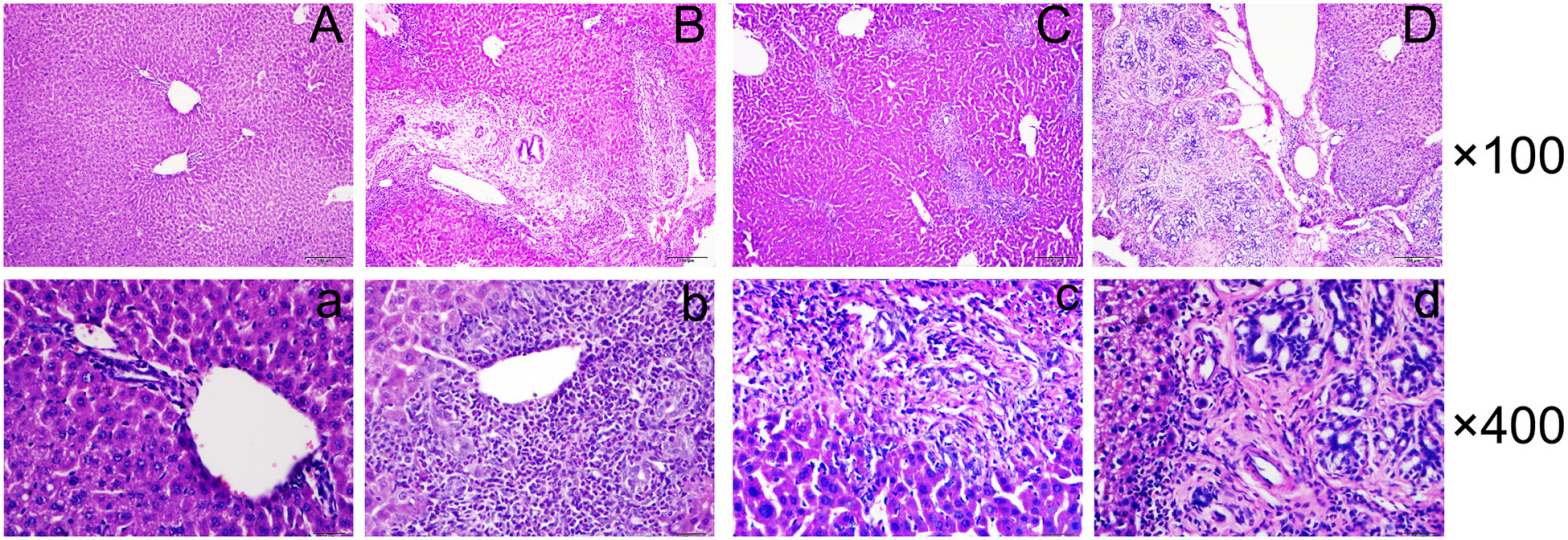
Representative histology of liver sections stained with hematoxylin and eosin (H&E). **A** and **a** show liver sections from NC mice. In infected mice, massive inflammatory cells were infiltrated around the portal areas accompanied by cholangiocyte proliferation on day 14 post-infection (PI). (**B** and **b**). The numbers of inflammatory cells decreased, but bile duct hyperplasia was exaggerated on day 28 PI. (**C** and **c**) and 56 PI. (**D** and **d**). Capitals and lowercase letters indicate magnifications of ×100 and ×400 times respectively.

**Fig 5:**
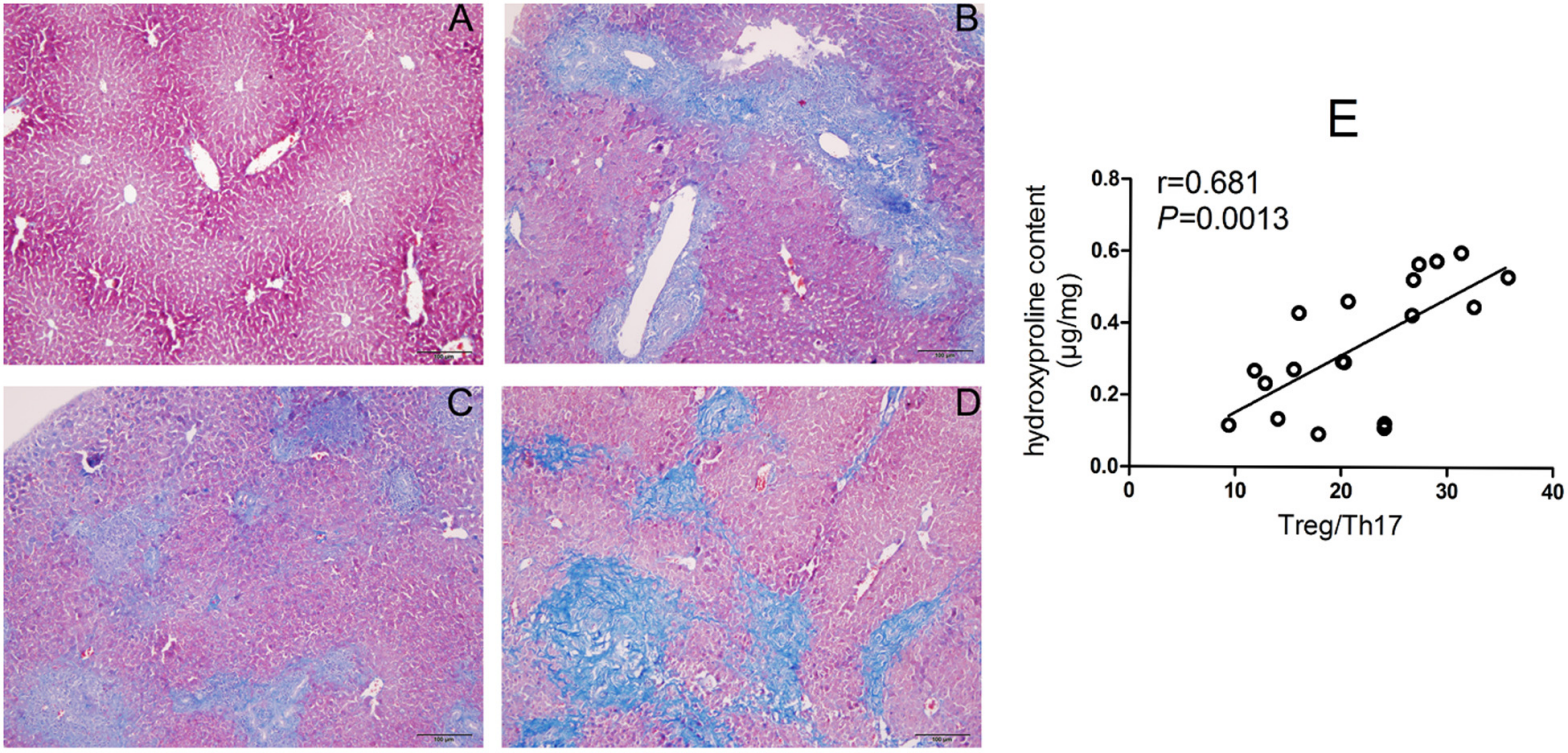
Collagen depositions in the liver of *Clonorchis sinensis*-infected BALB/c mice. Compared to NC mice (**A**), collagen fibers were slightly stained blue by Masson’s trichrome staining on day 14 post-infection (PI) (**B**), and the degree of staining were increased on day 28 PI (**C**) and 56 PI (**D**). Correlations were calculated between the degree of Treg/Th17 imbalance and liver fibrosis caused by *C*. *sinensis* (**E**).

Hematoxylin and eosin (H&E) and Masson’s trichrome staining were employed to explore the potential relationship between Treg/Th17 imbalance and histologic changes in the liver of *C*. *sinensis*-infected mice. As shown in Fig 4, substantial numbers of inflammatory cells had infiltrated around the portal area, collagen depositions were slightly stained, and intrahepatic bile duct epithelia were proliferated in the liver tissue, and these changes were accompanied by hepatocellular necrosis on day 14 PI (Fig 4A and 4a). The intrahepatic bile ducts continued to be exaggerated extensively on day 28 PI and 56 PI. (Fig 4C and 4D). However, inflammatory cell infiltration appeared to be attenuated (Fig 4C, 4D, 4c and 4d), whereas collagen deposition was extensively stained in the portal area of the liver in infected mice on day of 28 and 56 PI (Fig 5C and 5D). The result also showed that there was a positive correlation between the shifted Treg/Th17 paradigm and hydroxyproline content in the livers of infected mice (r = 0.681, *P*<0.001, Fig 5E), suggesting that the Treg/Th17 imbalance might be partly responsible for *C*. *sinensis*-induced liver fibrosis.

## Discussion

In the present study, we investigated dynamic changes in Th17 and Treg percentages and analyzed the potential effects of their imbalance on disease outcomes in BALB/c mice. We and others have demonstrated that this strain is relatively susceptible to *C*. *sinensis* infection compared with ICR, DDY, CBA/N, C57BL/6, and C3H/HeN mice [30–32]. However, the data obtained here revealed that there was no difference between egg-positive and egg-negative mice (data not shown), suggesting that the low egg-positive rate in the present study was likely due to the low sensitivity of assay used. It is impossible to detect eggs on day 14 PI as *C*. *sinensis* matures on day 23.2 PI on average [30]; however, histopathologic changes were already observed in all *C*. *sinensis*-infected mice on day 14 PI. These results indicate successful establishment of the mouse model of *C*. *sinensis* infection in the present study.

Similar to infection with other helminths, the immune response to *C*. *sinensis* is characterized by Th2-dominated responses that may contribute to the development of chronic infection and the host’s inability to clear the parasites [33–34]. CD4^+^ T cells are considered to play a pivotal role in orchestrating host immune response through the secretion of various cytokines [34–36]. However, the effects of dynamic Treg/Th17 changes on clonorchiasis are unknown. We investigated these changes in *C*. *sinensis*-infected mice. Our results show that only Treg in splenic CD4^+^ T cells were suppressed on day 14 PI, but Th17 and Treg were statistically increased in the liver and spleen on day 56 PI (Figs 1B, 1C, 2A and 2B) compared with NC mice. Importantly, we also observed increases in the Treg/Th17 ratio on day 28 and 56 PI in BALB/c mice infected with 45 *C*. *sinensis* metacercariae. We performed histologic examinations at different stages of infection to explore the potential relationships between histologic changes and Treg/Th17 imbalance. Our results showed an appropriate balance between Th17 and Treg during early infection (14 days PI), but inflammatory cells were recruited around the infection site at that time. Once the balance of Treg/Th17 was disturbed, the quantity of inflammatory cells was reduced, and collagen depositions sharply increased as the infection developed [27]. Thus, our earlier and present results suggest that the impaired balance between Treg and Th17 during *C*. *sinensis* infection might play a role in the development of liver fibrosis. Our results are in line with previous findings that the imbalance between Treg and Th17 can positively affect hepatic stellate cell activation and liver fibrosis development [37–38].

Cytokines play a significant role in facilitating the differentiation of naive CD4^+^ T cells into T helper (Th1, Th2, Th9, and Th17) and Treg cells [23]. In general, IL-6 combined with TGF-β can induce the differentiation of Th17 cells from naïve CD4^+^T cells, which can also be polarized into Treg cells following co-stimulation with IL-2 and TGF-β [20,39–40]. Previous studies have demonstrated that IL-6 plays a pivotal role in regulating the balance between Treg and Th17; it can induce the development of Th17 and restrain TGF-β-induced differentiation of Treg [41–42]. Overall, these findings are in agreement with our study. For example, the Treg percentage decreased when IL-6 concentration peaked during early infection (14 days PI), indicating that a high concentration of this cytokine might suppress Treg development. As the infection developed, the ratio of Treg increased, the level of IL-6 decreased to a normal level, and IL-2 sharply increased. This suggests that the negative effects of IL-6 on Treg were attenuated, and an increasing IL-2 concentration combined with TGF-β_1_ induced the polarization of Treg from naïve CD4^+^ T cells. In addition, the increased ratio of Treg to Th17 suggested that a peripheral tolerance was induced during *C*. *sinensis* infection and B cells which act as professional antigen-presenting cells may play a role in the induction of Tregs [43–45].

The percentage of Th17 steadily increased as the infection developed, suggesting that an alternative pathway polarizes Th17 cells in addition to the IL-6 and TGF-β_1_ signaling pathways [46–48]. As Th17 is involved in the immunopathology of infectious diseases and autoimmune diseases, our results implied that immune therapeutics could be an alternative therapy to overcome the immune evasion caused by C. sinensis, for example, treatment with GM-CSF which has profound capacities of regulation of the immune response and maintenance of immunological tolerance could potentially promote Tregs amounts and function, thus, it may alleviate the immunopathology caused by *C*. *sinensis* [49–52].

In conclusion, our findings provide the first evidence of dynamic changes in Treg/Th17 and confirm an imbalance of these cell populations in *C*. *sinensis*-infected mice. Additional studies are needed to elucidate the detailed roles of Treg and Th17 in the pathogenesis of clonorchiasis.

## Supporting Information

S1 Fig 1 Eggs recovered from the mice infected by Clonorchis sinensis. Feces from all infected mice were screened to detect *C*. *sinensis* eggs microscopically from day 25 to 56 post-infection. The egg (arrow) was photographed under a microscope with magnifications of ×100.
(TIF)
