# Supplementary figures and images for: The Dynamics of Treg/Th17 and the Imbalance of Treg/Th17 in Clonorchis sinensis-Infected Mice
Source: PLoS One. 2015 Nov 23;10(11):e0143217. doi: 10.1371/journal.pone.0143217 (PMC4658164; doi:10.1371/journal.pone.0143217)

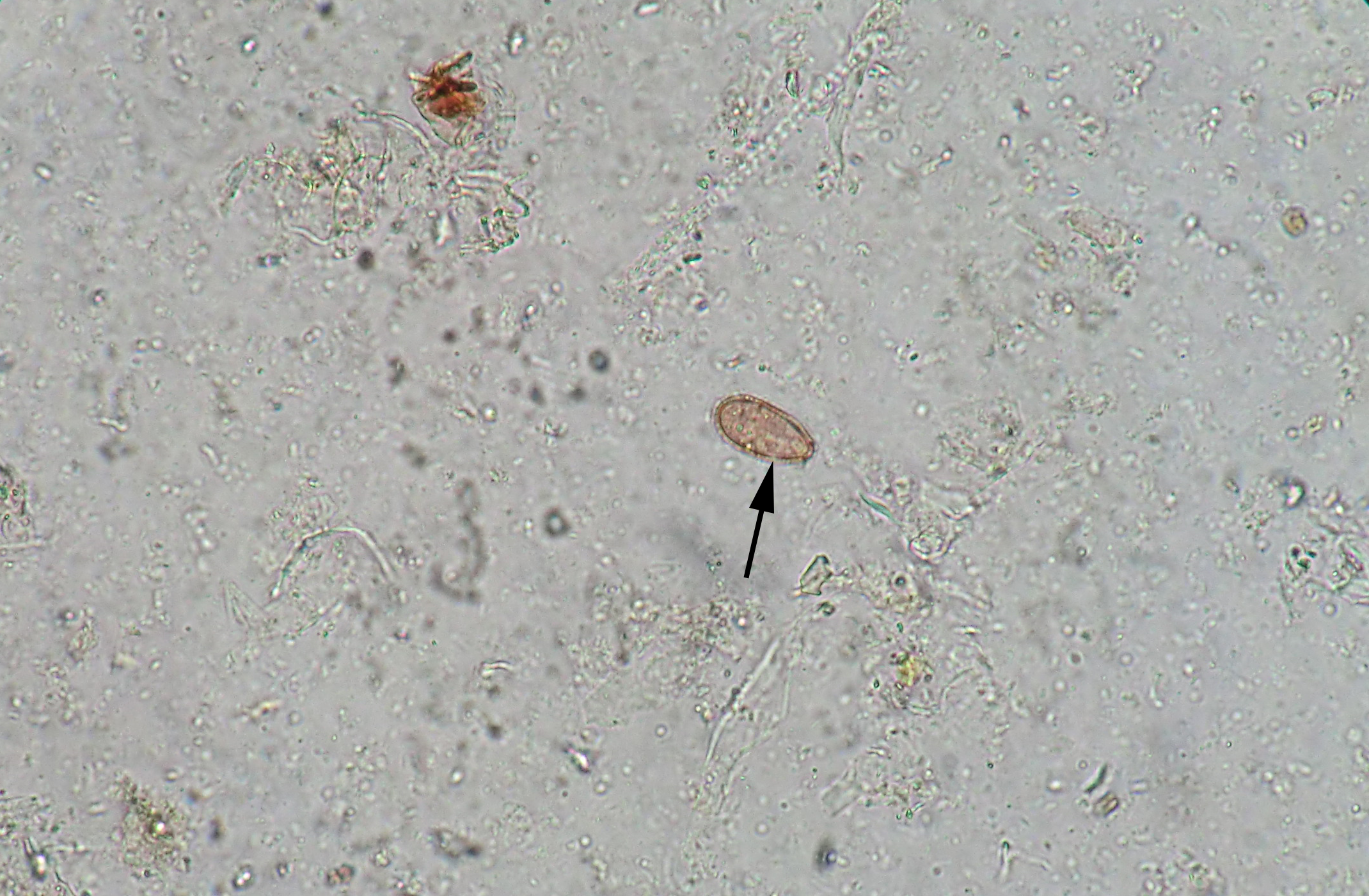

Supplement: S1 Fig — Feces from all infected mice were screened to detect C. sinensis eggs microscopically from day 25 to 56 post-infection. The egg (arrow) was photographed under a microscope with magnifications of ×100. (TIF) [file pone.0143217.s001.tif]
